# The relationship between self-compassion, coping style, sleep quality, and depression among college students

**DOI:** 10.3389/fpsyg.2024.1378181

**Published:** 2024-06-07

**Authors:** Yiwen Wang, Tiantian Fu, Jun Wang, Shufeng Chen, Guoxiao Sun

**Affiliations:** School of Physical Education, Shandong University, Jinan, Shandong, China

**Keywords:** self-compassion, coping style, sleep quality, depression, college students

## Abstract

**Background:**

The prevalence of sleep quality problems and depression in the college student population has attracted widespread attention. However, the factors influencing this are still unclear. The objective of this study was to investigate the associations between self-compassion (S-C), sleep quality (SQ), and depression (DEP) among college students and examine the mediating effects of coping style (CS) between the variables.

**Methods:**

A total of 1,038 Chinese university students were recruited for the study. The study used the Self-Compassion Scale (SCS), Simplified Coping Style Questionnaire (SCSQ), Depression Anxiety Stress Scale 21 (DASS-21), and Pittsburgh Sleep Quality Index (PSQI) to conduct the survey.

**Results:**

The self-compassion and coping style showed significant negative correlations with sleep quality and depression. Coping style partially mediated the relationship between self-compassion and sleep quality. The coping style also fully mediated the relationship between self-compassion and depression.

**Conclusion:**

This study reveals the associations between self-compassion and sleep quality and depression, and the mediating role of coping style among college students. This study provides valuable insights for improving sleep quality and alleviating depression problems among college students. It emphasizes the importance of self-compassion and positive coping style.

## Introduction

Sleep and mental health are common problems among college students. Sleep problems are prevalent among college students, while depression has a relatively high prevalence among college students. Depression and sleep quality are closely associated with the mental health and quality of life of college students. Depression and sleep quality were closely associated with the mental health and quality of life of college students. According to statistics, college students have increasingly prominent sleep problems, and the detection rate of sleep problems is as high as 62% (Becker et al., 2018). Chronically poor sleep quality may lead to anxiety and self-injurious behaviors (Adams and Kisler, 2013; Khazaie et al., 2021). The World Health Organization predicts that depression will become the primary contributor to the global burden of disease by 2030 (World Health Organization, 2019). Therefore, this study aimed to focus on sleep quality and depression among college students.

The development of depression and the decline in sleep quality are closely related to the individual’s cognitive style. In psychology, self-compassion is considered a psychological trait related to emotion regulation and coping. Self-compassion may have an important role in depression and sleep problems. Self-compassion is an emotionally positive self-attitude that could protect against the negative consequences of self-judgment, isolation, and rumination (Neff, 2003). Previous research has shown a negative correlation between self-compassion and sleep quality (Rakhimov et al., 2022). High levels of self-compassion may have a protective effect on sleep quality (Kemper et al., 2015). Individuals with high self-compassion tendencies are more inclined to maintain regular sleep and get enough sleep because they are more likely to cope positively, reducing insomnia (Neff, 2003; Sirois et al., 2015). In addition, self-compassion interventions have been shown to significantly improve the participants’ sleep quality (Butz and Stahlberg, 2018). On the other hand, there was a significant correlation between self-compassion and depression (Biskas et al., 2022). Depression refers to loss of interest, negative self-evaluation, and social obstacles, which seriously affect mental health and quality of life (Beck, 1961). Individuals who lack self-compassion are more likely to experience depressive symptoms (Hood et al., 2020). According to cognitive theory, a lack of self-compassion may lead to negative emotions and self-denial, further affecting the development of depression (Beck, 1964). Self-kindness and mindfulness are important components of self-compassion, and they can help individuals remain caring and accepting of themselves, which can alleviate depression (Kuyken et al., 2010; Joeng et al., 2017; Kurebayashi, 2020; Zhou et al., 2022). In conclusion, studies have shown significant correlations between self-compassion and sleep quality. There was also a significant correlation between self-compassion and depression. Self-compassion is a positive psychological variable that may play an important role in alleviating depression and promoting sleep quality.

The decline in sleep quality and the development of depression are influenced by the individual’s cognitive style. Coping style is a cognitive style that refers to how individuals are accustomed to dealing with various stressors (Pearlin and Schooler, 1978). On the one hand, the coping style was associated with sleep quality (Gargiulo et al., 2021). According to the psycho-bio-behavioral model of insomnia vulnerability, coping style was an important factor affecting sleep quality (Harvey et al., 2014; Ellis et al., 2021). Previous research has established that a positive coping style was associated with better sleep quality (Kim et al., 2022). In a prospective cohort study, the inappropriate coping style led to sleep problems (Otsuka et al., 2022). Besides, the cognitive-depression model suggested that negative cognition may lead to depression (Beck, 1970). Lower levels of self-compassion and negative coping styles are both negative perceptions. The absence of self-compassion may result in cognitive distortions and the development of depression. Individuals may approach situations objectively when coping positively, whereas those who adopt a negative coping style may experience depression (Clarke and Goosen, 2009; Sun et al., 2017; Wu et al., 2020).

On the other hand, research has shown that self-compassion is critical to coping style (Huang et al., 2021 Li et al., 2021). Self-compassion was considered a positive psychological variable that promotes a positive coping style (Ewert et al., 2021). Individuals who have a high level of self-compassion are more likely to use a positive coping style (Keyes et al., 2023). Positive coping style was associated with a lower prevalence of sleep problems (Ren et al., 2021). Conversely, individuals with lower self-compassion scores exhibited a greater propensity to adopt a negative coping style (Beato et al., 2021), which may lead to poor sleep quality (Hagemann et al., 2023). Besides, positive coping style help alleviate depression and reduce the impact of negative emotions. Individuals who employ a positive coping style may report lower depression (Allman et al., 2009). In conclusion, self-compassion can motivate individuals to adopt positive coping style to effectively face setbacks and failures. Negative coping style may lead to depression and decreased sleep quality.

**Figure 1:**
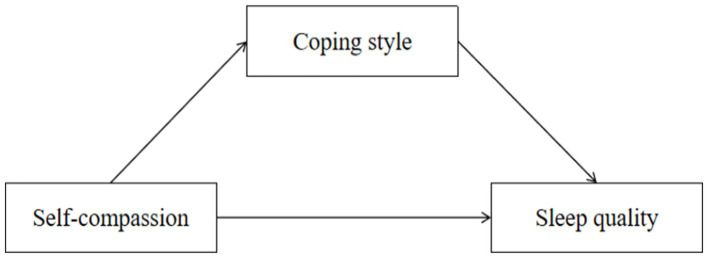
Conceptual model 1 (M1).

**Figure 2:**
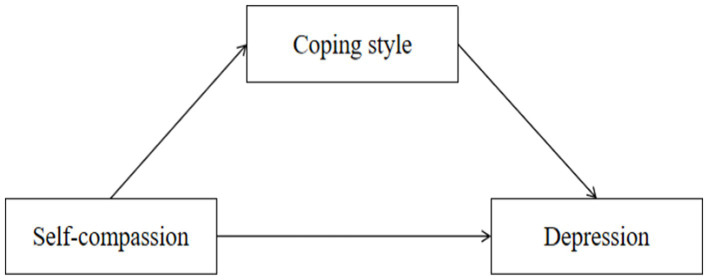
Conceptual model 2 (M2).

The objective of this study was to examine the effects of self-compassion and coping style on sleep quality and depression among college students. We established the following Hypothesis: H1: Self-compassion is negatively associated with sleep quality; H2: Self-compassion is negatively associated with depression; H3: Coping style plays a mediating role between self-compassion and sleep quality; and H4: Coping style plays a mediating role between self-compassion and depression. The structural models are shown in Figures 1, 2.

## Methods

### Participants and procedures

We used an electronic questionnaire, including a short letter explaining the motivation for the study and emphasizing the confidentiality and anonymity of this study. We collected 1,506 questionnaires covering basic sociodemographic information, as well as data on self-compassion, coping style, sleep quality, and depression. After the questionnaires were returned, we performed quality control on those with 100% completion. We excluded invalid questionnaires that were filled out indiscriminately or had consecutively repeated answers, resulting in 1038 questionnaires with a validity rate of 86.85%. The study population consisted of 1,038 college students (male = 734, female = 304; M_age_ = 23, SD = 2.714), mainly from six universities in China. Specific information about the participants is presented in Table 1. This study (20190912) was approved by the Public Health Ethics Committee of Cheelu College of Medicine, Shandong University.

**Table 1:** Demographic data (*N* = 1,038).

Variables	Category	*N*	%
Gender	Male	734	70.70
	Female	304	29.30
Residence	Rural	769	74.10
	Town	269	25.90
Grade	Freshman	40	3.90
	Sophomore	354	34.10
	Junior	397	38.20
	Senior	176	17.00
	Graduate student	71	6.80
Major	Liberal arts	316	30.40
	Sciences	572	55.10
	Engineering	118	11.40
	Medicine	32	3.10
Only child	Yes	642	61.80
	No	396	38.20

### Measures

#### Self-compassion scale (SCS)

This study used the self-compassion scale compiled by Neff (2003). The scale consists of 26 items, including six dimensions. Among them, 13 items are reverse-scoring questions. The responses used a 5-point Likert scale where “never” =1, “seldom” =2, “sometimes” =3, “most of the time” =4, and “always” =5. The higher score represents a higher level of self-compassion. The reliability and validity of this scale were good in previous studies (Hirsch et al., 2021). The Cronbach’s α coefficient for the scale in this study was 0.832.

#### Simple coping style questionnaire (SCSQ)

This study adopted the questionnaire compiled by Xie (1998), which is about the different attitudes and measures people may take in dealing with life events in daily life. The subscale included 20 items. It is a 4-point Likert-type scale. This study adopted the individual coping style tendency formula. The coping tendency score is the standardized score of positive coping minus the standardized score of negative coping. If the coping tendency value is greater than 0, it means that people adopt positive coping; if it is less than 0, it means that people adopt negative coping (Dai et al., 2015). Xie (1998) has validated the reliability and validity of this scale. The Cronbach’s α coefficient for the scale in this study was 0.951.

#### DASS-21 depression subscale

This study used the Depression subscale of the Depression-Anxiety-Stress Scale 15.6 (Lovibond and Lovibond, 1995). We tested depression scores using the depression subscale. The depression subscale included seven items. The responses used a 4-point Likert scale where “never” =1, “sometimes” =2, “most of the time” =3, and “always” =4. As the score increases, there is a corresponding elevation in the level of depression. The Cronbach’s α coefficient for the depression subscale was 0.925.

#### Pittsburgh sleep quality index (PSQI)

This study used the Pittsburgh Sleep Quality Index scale (Buysse et al., 1989). This scale measures the participants’ sleep quality in the last month, consisting of 19 self-assessments and five other assessment items. The corresponding scores mainly include sleep quality, time, efficiency, disorders, hypnotic drug use, and daytime dysfunction. The PSQI score is divided into three grades: PSQI ≤4 is good, 5 ≤ PSQI ≤7 is medium, and PSQI ≥8 is poor. In this study, the Chinese version was revised by Liu et al. (1996), which had good reliability and validity, test–retest reliability, construct validity, and empirical validity. The scale showed a Cronbach’s α coefficient of 0.84.

### Statistical analysis

This study used Excel for initial data collation, and IBM SPSS Statistics, version 26, and PROCESS v4.1 were used for descriptive statistics, correlation analysis, and regression analysis (Hayes and Rockwood, 2017). Descriptive statistics were used to report each variable’s mean and standard deviation. We utilized Pearson correlation analysis to examine the relationship between variables. The study used the Model 4 of PROCESS to test the hypothesis and investigate the mediating effect. In PROCESS, a Bootstrap sample of 2,000 was selected, and 95% confidence intervals (*CIs*) were calculated. The parameter estimations were significant if the 95% *CIs* did not contain 0 (Wen et al., 2004).

## Results

### Common method Bias analysis

Using self-reporting to collect data may lead to common method bias. The study used a procedural control method during the measurement process to reduce the impact of common method bias. The questionnaire contains appropriate reverse-scoring questions and randomly arranges the order of the questionnaire. Before the questionnaire was issued, the subjects were informed to fill out the questionnaire anonymously. The results are confidential and only used for academic research. In addition, the Harman single-factor test was used (Zhou and Long, 2004). By conducting a principal component analysis, we identified nine components that exhibited eigenvalues greater than one. The first factor was found to explain 36.164% of the variation, which was less than the required criterion of 40%. It demonstrates that there is no common method bias in this study (Podsakoff et al., 2003).

### Demographic variables

This study collected information such as gender, age, grade, major, place of residence, and whether they were only children. Grades were divided into freshman, sophomore, junior, senior, or senior five and graduate students, and majors were divided into liberal arts, science, engineering, and medicine; The residence can be divided into urban and rural areas. This study investigated 1,038 participants with a mean age of 23 years (*SD* = 2.714). The specific data information is presented in Table 1.

### Descriptive statistics and correlation analysis

The self-compassion score was 70.380 ± 6.600, and the sleep quality and depression scores were 10.020 ± 4.180 and 21.570 ± 4.504. The coping style tendency was greater than 0 means that college students tend to adopt a positive coping style. Self-compassion was significantly negatively associated with sleep quality (*r* = −0.514, *p* < 0.01) and depression (*r* = −0.216, *p* < 0.01). Besides, self-compassion positively correlated with coping style (*r* = 0.606, *p* < 0.01). In addition, the coping style was significantly negatively correlated with sleep quality (*r* = −0.453, *p* < 0.01) and depression (*r* = −0.410, *p* < 0.01). Finally, there was a significantly positive correlation between depression and sleep quality (*r* = 0.494, *p* < 0.01). The results are shown in Table 2.

**Table 2:** Descriptive statistics and correlation analysis for the variables (*N* = 1,038).

Variables	*M*	SD	1	2	3	4
Self-compassion	70.380	6.600	1.000	-	-	-
Coping style	0.010	0.810	0.606^**^	1.000	-	-
Sleep Quality	10.020	4.180	−0.514^**^	−0.453^**^	1.000	-
Depression	21.570	4.540	−0.216^**^	−0.410^**^	0.494^**^	1.000

M, Mean; SD, Standard Deviation; ^**^p < 0.01.

### Regression analysis

The results presented the mediating regression coefficients for coping style as a mediating variable between self-compassion to sleep quality and depression. Self-compassion was significantly and positively correlated with coping style (*β* = 0.497, *p* < 0.001). Self-compassion (*β* = −0.378, *p* < 0.001) and coping style (*β* = −0.222, *p* < 0.001) were significantly and negatively correlated with sleep quality. Besides, coping style was significantly negatively correlated with depression (*β* = −0.440, *p* < 0.001). Specific results are presented in Table 3.

**Table 3:** The results of the regression estimate of the mediation (*N* = 1,038).

Outcome Variables	Predictors	Goodness-of-fit indices			Regression coefficient and significance	
		*R*	*R* ^2^	*F*	β	*t*
CS	S-C	0.606	0.367	602.601^***^	0.497	24.547^***^
SQ	S-C	0.543	0.295	217.170^***^	−0.378	−11.545^***^
	CS				−0.222	−6.794^***^
DEP	S-C	0.411	0.169	105.639^***^	0.051	1.432
	CS				−0.440	−12.370^***^

S-C, Self-compassion; CS, Coping Style; DEP, Depression; SQ, Sleep Quality; ^***^p < 0.001, ^*^p < 0.05.

### Mediation analysis

**Figure 3:**
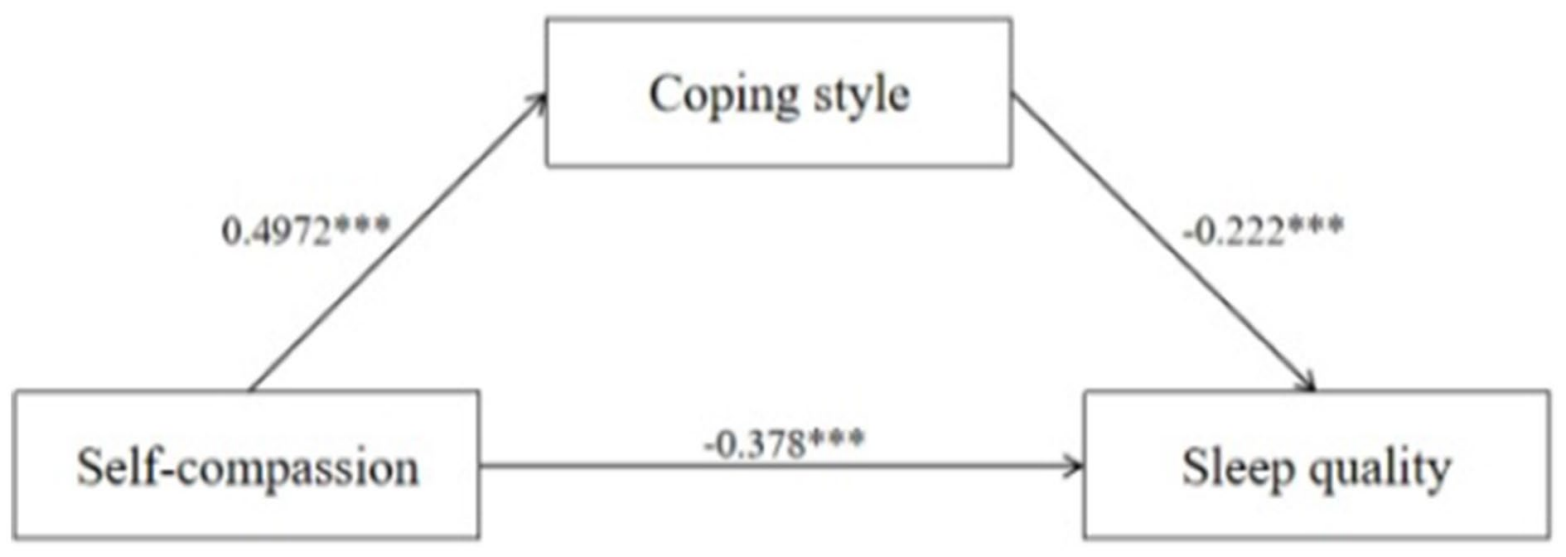
Mediation effect analysis of M1 (^***^p < 0.001).

**Figure 4:**
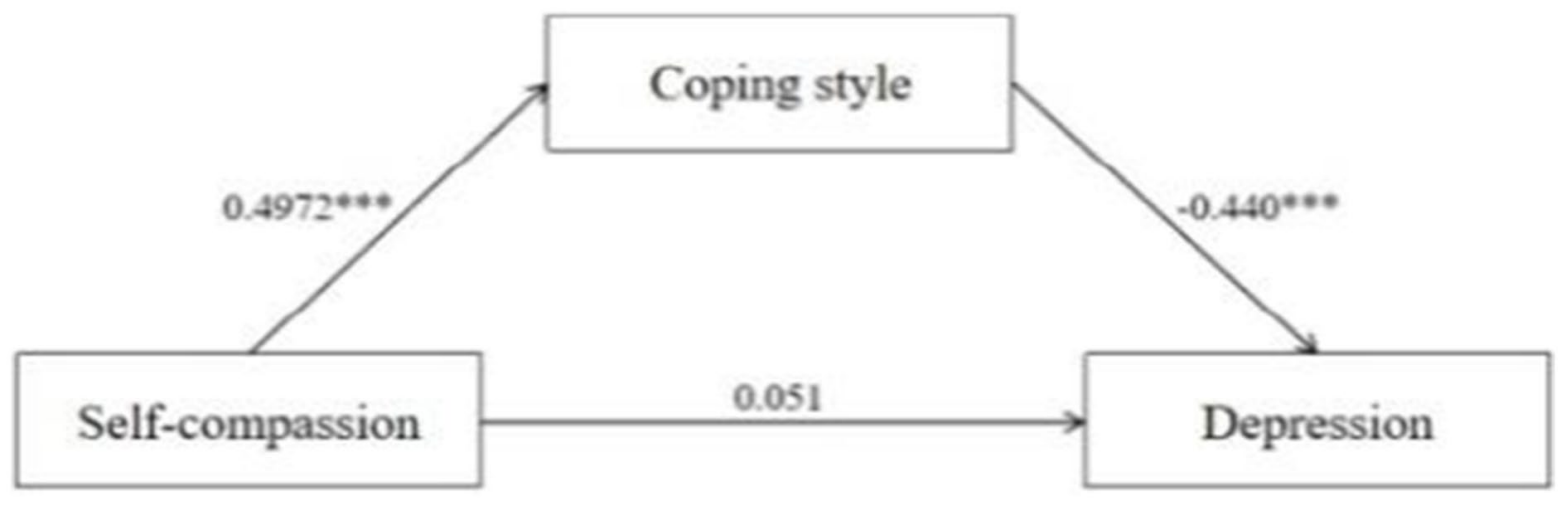
Mediation effect analysis of M2 (^***^p < 0.001).

To test the pathways above, we employed the bias-corrected percentile Bootstrap method with a self-sampling size of 2,000. Additionally, we calculated 95% confidence intervals (*CIs*). Both direct and indirect effects between self-compassion and sleep quality were significant. Coping style mediated the relationship between self-compassion and sleep quality. Besides, there was no significant direct effect observed between self-compassion and depression, while the indirect effect was found to be significant. The coping style acted as a mediator between self-compassion and depression. Table 4 and Figures 3, 4 show the mediation analysis results.

**Table 4:** Bootstrap analysis of the test for mediating effects (*N* = 1,038).

Model	Effect types	Path	Effect	SE	Bootstrap 95%CI
Model 1	Direct effect	S-C → SQ	−0.378	0.027	−0.443 to −0.314
	Indirect effect	S-C → CS → SQ	−0.135	0.232	−0.182to −0.090
	Total effect		−0.514	0.026	−0.566 to −0.461
Model 2	Direct effect	S-C → DEP	0.051	0.035	−0.018 to 0.120
	Indirect effect	S-C → CS → DEP	−0.267	0.031	−0.333 to −0.208
	Total effect		−0.216	0.030	−0.275 to −0.156

S-C, Self-compassion; CS, Coping Style; DEP, Depression; SQ, Sleep Quality.

## Discussion

This study examined that self-compassion and coping style have significant correlations with sleep quality and depression among college students. The mediating role of coping style among college students was validated and highlighted. We discovered that: (1) self-compassion and coping style showed significant negative correlations with sleep quality and depression. (2) coping style partially mediated the relationship between self-compassion and sleep quality. (3) coping style fully mediated the relationship between self-compassion and depression.

Based on our findings, we found that self-compassion showed a significant negative correlation with both sleep quality and depression. These two results support our hypothesis 1 and hypothesis 2. This is similar to previous studies, suggesting that individuals with higher levels of self-compassion may have lower levels of depression and better sleep quality (Hwang et al., 2019; Kurebayashi, 2020; Liu et al., 2023). Based on the self-regulation resource model of health behavior, self-compassion may promote healthy living behaviors such as sleep through adaptive emotions (Sirois et al., 2015). Specifically, self-compassion has the potential to act as a safeguard or protective factor for individual emotional regulation and coping abilities, thereby safeguarding sleep quality. Previous research has also found positive thoughts and self-friendliness to be adaptive emotions that promote healthy behaviors and protect sleep quality (Sirois et al., 2019). At the same time, high levels of self-compassion have a significant effect on depression (Wang et al., 2023). Therefore, self-compassion may be a key factor in sleep quality and have some potential impact on depression. We may consider interventions for self-compassion to enhance sleep quality and improve depression among college students.

Secondly, we found that coping style mediated the relationship between self-compassion and sleep quality. The result supports our hypothesis 3. It is important to understand how individuals cope with stress and maintain sleep quality. According to the Coping Interaction Model, individual differences in coping choices are the result of a combination of personality traits, individual differences, and stressful situations (Ye and Shen, 2002). Our findings suggest that individuals who exhibit higher levels of self-compassion were more inclined to adopt a positive coping style when confronted with stress (Ewert et al., 2021). This could be because individuals with elevated levels of self-compassion tend to be more mindful and attentive towards their emotions and needs. Therefore, they were more likely to adopt a positive style to cope with stress and difficulties. This positive coping style may help to relieve stress and promote good quality sleep (Li et al., 2021). Conversely, individuals with low levels of self-compassion may develop feelings of isolation, and negative cognitive perceptions and adopt a negative coping style (Neff, 2003; Li et al., 2021). This negative coping style may exacerbate sleep problems reducing the quality of sleep. Therefore, we suggest that we can try to regulate emotions and guide coping style through CBT (Cognitive behavioral therapy) to reduce negative cognition and behavior (Beck, 1970). A previous study has demonstrated that CBT is beneficial in treating insomnia and chronic fatigue syndrome (Drerup et al., 2021). It may be possible to use CBT training to improve sleep quality in college students.

Thirdly, we found that coping style mediated the relationship between self-compassion and depression, which supports our hypothesis 4. There is a strong correlation between self-compassion, coping style, and depression (Saalwirth and Leipold, 2021; Zhang et al., 2021; Gerace, 2022; Mushquash and Grassia, 2022). Individuals with negative cognition may adopt a negative coping style, which may lead to depression (Zheng et al., 2020). According to the cognitive depression model, negative cognitions may lead to depression (Beck, 1964). The present study validated the model and added coping style as a mediating variable. The results of the study showed that the level of self-compassion was negatively correlated with the level of depression. It suggests that an individual’s perceptions have a potential impact on mood. The results showed that coping style played a fully mediating role between self-compassion and depression. This suggests that coping styles are associated with depression levels and have an important role in the effect of depression. Especially among college students, when their levels of self-compassion are low, they may adopt a negative coping style. Negative cognition and coping style may exacerbate depression. The individual’s coping style may play an important role in depression. Therefore, we can develop self-compassion and relieve depression through the MBSR (Mindfulness-based stress reduction) (Kabat-Zinn, 2003; Chiesa and Serretti, 2009; Goldin and Gross, 2010). We suggest that developing self-compassion and instructing college students to adopt positive coping style reduce depression.

In conclusion, this study is significant. Future research could intervene by combining the following methods. We suggest that effective interventions can be provided to college students by fostering self-compassion and guided coping style, combined with MBSR and CBT training. This will help to improve sleep quality, reduce depression, and promote mental health. When improving depression, we can focus on providing positive guidance to college students. The theoretical significance of this study is that it validates the mediating role of coping style. This provides important clues and evidence for understanding the mechanisms of mental health problems among college students. In terms of practical implications, this study provides effective interventions for college students’ mental and physical health problems. By fostering self-compassion and guiding appropriate coping style, college students to reduce depression levels and improve sleep quality, thus promoting their health. This is important for the management of college students’ physical and mental health, which can help them improve their academic performance and promote their personal development. In addition, by proposing MBSR and CBT as interventions, it provides practical guidance for the field of mental health research and ideas for developing effective coping strategies and treatment programs.

## Limitation

This study has a few limitations that should be taken into account. Firstly, the findings are based on a cross-sectional design, which means that we cannot establish causal relationships between self-compassion, coping style, depression, and sleep quality. To address this limitation, future research should consider using longitudinal studies or experimental designs to examine these relationships over time. Secondly, the use of self-report measures to assess variables in this study introduces the possibility of biases, such as social desirability bias or recall bias. Lastly, it is important to acknowledge that the study sample comprised only college students. Consequently, the generalizability of the findings to other populations is limited.

## Conclusion

In summary, the results of this study reveal the relationship between self-compassion and sleep quality, and depression. Also, the mediating role of coping styles among college students was verified. The results of the study indicate that there is a significant negative relationship between self-compassion with sleep quality and depression. Specifically, coping style mediates the relationship between self-compassion and sleep quality. Coping style completely mediates the relationship between self-compassion and depression. We suggest that future research focus on developing self-compassion in college students and guiding them to choose appropriate coping style to improve sleep quality and reduce depression. These results have important practical implications for helping college students promote good sleep and maintain their mental health.

## Data availability statement

The raw data supporting the conclusions of this article will be made available by the authors, without undue reservation.

## Ethics statement

The studies involving humans were approved by Public Health Ethics Committee of Cheelu College of Medicine, Shandong University. The studies were conducted in accordance with the local legislation and institutional requirements. The participants provided their written informed consent to participate in this study. Written informed consent was obtained from the individual(s) for the publication of any potentially identifiable images or data included in this article.

## Author contributions

YW: Conceptualization, Data curation, Investigation, Methodology, Validation, Writing – original draft. TF: Formal analysis, Methodology, Software, Supervision, Validation, Writing – review & editing. JW: Investigation, Methodology, Supervision, Validation, Writing – review & editing. SC: Conceptualization, Investigation, Methodology, Software, Validation, Writing – review & editing. GS: Conceptualization, Data curation, Formal analysis, Funding acquisition, Investigation, Methodology, Project administration, Resources, Software, Supervision, Validation, Writing – review & editing.

## References

[ref1] Adams S. K. Kisler T. S. (2013). Sleep quality as a mediator between technology-related sleep quality, depression, and anxiety. Cyberpsychol. Behav. Soc. Netw. 16, 25–30. doi: 10.1089/cyber.2012.0157, PMID: 23320870

[ref2] Allman E. Berry D. Nasir L. (2009). Depression and coping in heart failure patients a review of the literature. J. Cardiovasc. Nurs. 24, 106–117. doi: 10.1097/JCN.0b013e318197a985 19242276

[ref3] Beato A. F. da Costa L. P. Nogueira R. (2021). ‘Everything is Gonna be alright with me’: the role of self-compassion, affect, and coping in negative emotional symptoms during coronavirus quarantine. Int. J. Environ. Res. Public Health 18:2017. doi: 10.3390/ijerph18042017, PMID: 33669661 PMC7923103

[ref4] Beck A. T. (1961). An inventory for measuring depression. Arch. Gen. Psychiatry 4:561. doi: 10.1001/archpsyc.1961.01710120031004 13688369

[ref5] Beck A. (1964). Thinking and depression: II. Theory and therapy. Arch. Gen. Psychiatry 10:561. doi: 10.1001/archpsyc.1964.01720240015003 14159256

[ref6] Beck A. (1970). Cognitive therapy—nature and relation to behavior therapy. Behav. Ther. 1, 184–200. doi: 10.1016/S0005-7894(70)80030-2 27993332

[ref7] Becker S. P. Jarrett M. A. Luebbe A. M. Garner A. A. Burns G. L. Kofler M. J. (2018). Sleep in a large, multi-university sample of college students: sleep problem prevalence, sex differences, and mental health correlates. Sleep Health 4, 174–181. doi: 10.1016/j.sleh.2018.01.001, PMID: 29555131 PMC5863586

[ref9] Biskas M. Sirois F. M. Webb T. L. (2022). Using social cognition models to understand why people, such as perfectionists, struggle to respond with self-compassion. Br. J. Soc. Psychol. 61, 1160–1182. doi: 10.1111/bjso.12531, PMID: 35262948 PMC9790291

[ref10] Butz S. Stahlberg D. (2018). Can self-compassion improve sleep quality via reduced rumination? Self Identity 17, 666–686. doi: 10.1080/15298868.2018.1456482

[ref11] Buysse D. Reynolds C. Monk T. Berman S. Kupfer D. (1989). The Pittsburgh sleep quality index—a new instrument for psychiatric Practice and research. Psychiatry Res. 28, 193–213. doi: 10.1016/0165-1781(89)90047-4 2748771

[ref12] Chiesa A. Serretti A. (2009). Mindfulness-based stress reduction for stress Management in Healthy People: a review and Meta-analysis. J. Altern. Complement. Med. 15, 593–600. doi: 10.1089/acm.2008.0495, PMID: 19432513

[ref13] Clarke D. Goosen T. (2009). The mediating effects of coping strategies in the relationship between automatic negative thoughts and depression in a clinical sample of diabetes patients. Personal. Individ. Differ. 46, 460–464. doi: 10.1016/j.paid.2008.11.014

[ref14] Dai X. Zhang J. Cheng Z. Wang M. (2015). Handbook of commonly used psychological assessment scales. People’s Military Medical press.

[ref15] Drerup M. Roth A. Kane A. Sullivan A. B. (2021). Therapeutic approaches to insomnia and fatigue in patients with multiple sclerosis. Nat. Sci. Sleep 13, 201–207. doi: 10.2147/NSS.S256676, PMID: 33623461 PMC7896778

[ref16] Ellis J. G. Perlis M. L. Espie C. A. Grandner M. A. Bastien C. H. Barclay N. L.. (2021). The natural history of insomnia: predisposing, precipitating, coping, and perpetuating factors over the early developmental course of insomnia. Sleep 44:zsab095. doi: 10.1093/sleep/zsab095, PMID: 33849074 PMC8826168

[ref17] Ewert C. Vater A. Schroder-Abe M. (2021). Self-compassion and coping: a Meta-analysis. Mindfulness 12, 1063–1077. doi: 10.1007/s12671-020-01563-8

[ref18] Gargiulo A. T. Peterson L. M. Grafe L. A. (2021). Stress, coping, resilience, and sleep during the COVID-19 pandemic: a representative survey study of US adults. Brain Behav. 11:e2384. doi: 10.1002/brb3.2384, PMID: 34661981 PMC8613418

[ref19] Gerace A. (2022). Gentle gloves: the importance of self-compassion for mental health nurses during COVID-19. Int. J. Ment. Health Nurs. 31, 3–7. doi: 10.1111/inm.12934, PMID: 34580979 PMC8653245

[ref20] Goldin P. R. Gross J. J. (2010). Effects of mindfulness-based stress reduction (MBSR) on emotion regulation in social anxiety disorder. Emotion 10, 83–91. doi: 10.1037/a0018441, PMID: 20141305 PMC4203918

[ref21] Hagemann N. Kirtley O. J. Lafit G. Vancampfort D. Wampers M. Decoster J.. (2023). Coping and sleep quality in youth: an experience sampling study. J. Adolesc. 95, 566–583. doi: 10.1002/jad.12137, PMID: 36647754

[ref22] Harvey C.-J. Gehrman P. Espie C. A. (2014). Who is predisposed to insomnia: a review of familial aggregation, stress-reactivity, personality and coping style. Sleep Med. Rev. 18, 237–247. doi: 10.1016/j.smrv.2013.11.004, PMID: 24480386

[ref23] Hayes A. F. Rockwood N. J. (2017). Regression-based statistical mediation and moderation analysis in clinical research: observations, recommendations, and implementation. Behav. Res. Ther. 98, 39–57. doi: 10.1016/j.brat.2016.11.001, PMID: 27865431

[ref24] Hirsch J. K. Hall B. B. Wise H. A. Brooks B. D. Chang E. C. Sirois F. M. (2021). Negative life events and suicide risk in college students: conditional indirect effects of hopelessness and self-compassion. J. Am. Coll. Heal. 69, 546–553. doi: 10.1080/07448481.2019.1692023, PMID: 31765290

[ref25] Hood C. O. Ross L. T. Wills N. (2020). Family factors and depressive symptoms among college students: understanding the role of self-compassion. J. Am. Coll. Heal. 68, 683–687. doi: 10.1080/07448481.2019.1596920, PMID: 30958756 PMC7085279

[ref26] Huang J. Lin K. Fan L. Qiao S. Wang Y. (2021). The effects of a self-compassion intervention on future-oriented coping and psychological well-being: a randomized controlled trial in Chinese college students. Mindfulness 12, 1451–1458. doi: 10.1007/s12671-021-01614-8

[ref27] Hwang Y.-S. Medvedev O. N. Kraegeloh C. Hand K. Noh J.-E. Singh N. N. (2019). The role of dispositional mindfulness and self-compassion in educator stress. Mindfulness 10, 1692–1702. doi: 10.1007/s12671-019-01183-x

[ref28] Joeng J. R. Turner S. L. Kim E. Y. Choi S. A. Lee Y. J. Kim J. K. (2017). Insecure attachment and emotional distress: fear of self-compassion and self-compassion as mediators. Personal. Individ. Differ. 112, 6–11. doi: 10.1016/j.paid.2017.02.048

[ref29] Kabat-Zinn J. (2003). Mindfulness-based interventions in context: past, present, and future. Clin. Psychol. Sci. Pract. 10, 144–156. doi: 10.1093/clipsy.bpg016

[ref30] Kemper K. J. Mo X. Khayat R. (2015). Are mindfulness and self-compassion associated with sleep and resilience in health professionals? J. Altern. Complement. Med. 21, 496–503. doi: 10.1089/acm.2014.0281, PMID: 26218885 PMC4523072

[ref31] Keyes J. Yankouskaya A. Panourgia C. (2023). Self-compassion, coping strategies and gender differences in psychology, counselling and psychotherapy practitioners during COVID-19: lessons learnt. Couns. Psychother. Res. 23, 1052–1062., PMID: 36247723 10.1002/capr.12574 PMC9537790

[ref32] Khazaie H. Zakiei A. McCall W. V. Noori K. Rostampour M. Sadeghi Bahmani D.. (2021). Relationship between sleep problems and self-injury: a systematic review. Behav. Sleep Med. 19, 689–704. doi: 10.1080/15402002.2020.1822360, PMID: 32991212

[ref33] Kim S. M. Um Y. H. Kim T. W. Seo H.-J. Jeong J.-H. Hong S.-C. (2022). Mediation effect of the coping strategies on the relation between stress and sleep quality. Psychiatry Investig. 19, 580–587. doi: 10.30773/pi.2022.0015, PMID: 35903060 PMC9334809

[ref34] Kurebayashi Y. (2020). Effects of self-compassion and self-focus on sleep disturbances among psychiatric nurses. Perspect. Psychiatr. Care 56, 474–480. doi: 10.1111/ppc.12458, PMID: 31793686

[ref35] Kuyken W. Watkins E. Holden E. White K. Taylor R. S. Byford S.. (2010). How does mindfulness-based cognitive therapy work? Behav. Res. Ther. 48, 1105–1112. doi: 10.1016/j.brat.2010.08.003 20810101

[ref37] Li A. Wang S. Cai M. Sun R. Liu X. (2021). Self-compassion and life-satisfaction among Chinese self-quarantined residents during COVID-19 pandemic: a moderated mediation model of positive coping and gender. Personal. Individ. Differ. 170:110457. doi: 10.1016/j.paid.2020.110457, PMID: 33100455 PMC7576372

[ref38] Liu X. Tang M. Hu L. Wang A. Wu H. Zhao G.. (1996). A reliability and validity study of the Pittsburgh sleep quality index. Chin. J. Psychiatry 2, 103–107.

[ref39] Liu A. Xu B. Liu M. Wang W. Wu X. (2023). The reciprocal relations among self-compassion, and depression among adolescents after the Jiuzhaigou earthquake: a three-wave cross-lagged study. J. Clin. Psychol. 79, 1786–1798. doi: 10.1002/jclp.23501, PMID: 36883442

[ref40] Lovibond P. Lovibond S. (1995). The structure of negative emotional states—comparison of the depression anxiety stress scales (dass) with the Beck depression and anxiety inventories. Behav. Res. Ther. 33, 335–343. doi: 10.1016/0005-7967(94)00075-U, PMID: 7726811

[ref43] Mushquash A. R. Grassia E. (2022). Coping during COVID-19: examining student stress and depressive symptoms. J. Am. Coll. Heal. 70, 2266–2269. doi: 10.1080/07448481.2020.1865379 33513079

[ref44] Neff K. (2003). Self-compassion: an alternative conceptualization of a healthy attitude toward oneself. Self Identity 2, 85–101. doi: 10.1080/15298860309032

[ref45] Otsuka Y. Itani O. Matsumoto Y. Kaneita Y. (2022). Associations between coping strategies and insomnia: a longitudinal study of Japanese workers. Sleep 45:zsab244. doi: 10.1093/sleep/zsab244, PMID: 34585730 PMC8842145

[ref46] Pearlin L. Schooler C. (1978). Structure of coping. J. Health Soc. Behav. 19, 2–21. doi: 10.2307/2136319 649936

[ref47] Podsakoff P. M. MacKenzie S. B. Lee J. Y. Podsakoff N. P. (2003). Common method biases in behavioral research: a critical review of the literature and recommended remedies. J. Appl. Psychol. 88, 879–903. doi: 10.1037/0021-9010.88.5.879, PMID: 14516251

[ref48] Rakhimov A. Ong J. Realo A. Tang N. K. Y. (2022). Being kind to self is being kind to sleep? A structural equation modelling approach evaluating the direct and indirect associations of self-compassion with sleep quality, emotional distress and mental well-being. Curr. Psychol. 42, 14092–14105. doi: 10.1007/s12144-021-02661-z

[ref49] Ren Z. Zhang X. Shen Y. Li X. He M. Shi H.. (2021). Associations of negative life events and coping styles with sleep quality among Chinese adolescents: a cross-sectional study. Environ. Health Prev. Med. 26:85. doi: 10.1186/s12199-021-01007-2, PMID: 34481463 PMC8418725

[ref50] Saalwirth C. Leipold B. (2021). Well-being and sleep in stressful times of the COVID-19 pandemic: relations to worrying and different coping strategies. Stress. Health 37, 973–985. doi: 10.1002/smi.3057, PMID: 33913244 PMC8237007

[ref51] Sirois F. M. Kitner R. Hirsch J. K. (2015). Self-compassion, affect, and health-promoting behaviors. Health Psychol. 34, 661–669. doi: 10.1037/hea0000158, PMID: 25243717

[ref52] Sirois F. M. Nauts S. Molnar D. S. (2019). Self-compassion and bedtime procrastination: an emotion regulation perspective. Mindfulness 10, 434–445. doi: 10.1007/s12671-018-0983-3

[ref53] Sun X. Niu G. You Z. Zhou Z. Tang Y. (2017). Gender, negative life events and coping on different stages of depression severity: a cross-sectional study among Chinese university students. J. Affect. Disord. 209, 177–181. doi: 10.1016/j.jad.2016.11.025, PMID: 27923194

[ref54] Wang S. Tang Q. Lv Y. Tao Y. Liu X. Zhang L.. (2023). The temporal relationship between depressive symptoms and loneliness: the moderating role of self-compassion. Behav. Sci. 13:472. doi: 10.3390/bs13060472, PMID: 37366723 PMC10294791

[ref55] Wen Z. Zhang L. Hou J. Liu H. (2004). Mediation effects test procedure and its application. Acta Psychol. Sin. 5, 614–620.

[ref56] World Health Organization. (2019), *Mental Disorder*. Available at: https://www.who.int/zh/news-room/fact-sheets/detail/mental-disorders

[ref57] Wu Y. Yu W. Wu X. Wan H. Wang Y. Lu G. (2020). Psychological resilience and positive coping styles among Chinese undergraduate students: a cross-sectional study. BMC Psychol. 8:79. doi: 10.1186/s40359-020-00444-y, PMID: 32762769 PMC7406959

[ref58] Xie Y. (1998). A preliminary study of the reliability and validity of the Brief Coping Style Scale. Chin. J. Clin. Psychol. 2, 53–54.

[ref59] Ye Y. Shen Y. (2002). Review of coping studies and coping style. J. Psychol. Sci. 6, 755–756. doi: 10.16719/j.cnki.1671-6981.2002.06.040

[ref60] Zhang Y. Huang L. Luo Y. Ai H. (2021). The relationship between state loneliness and depression among youths during COVID-19 lockdown: coping style as mediator. Front. Psychol. 12:701514. doi: 10.3389/fpsyg.2021.701514, PMID: 34594266 PMC8476915

[ref61] Zheng Z. Han W. Zhou Y. Zhang N. (2020). Childhood maltreatment and depression in adulthood in Chinese female college students: the mediating effect of coping style. Front. Psych. 11:581564. doi: 10.3389/fpsyt.2020.581564, PMID: 33240133 PMC7678483

[ref62] Zhou T. Bian X. Zhang K. Zheng S. Lin Y. Zheng H.. (2022). Maternal anxiety symptoms and Chinese adolescents’ mental health during the COVID-19 pandemic: the protective role of adolescents’ self-compassion. Front. Psych. 13:837846. doi: 10.3389/fpsyt.2022.837846, PMID: 35463484 PMC9026151

[ref63] Zhou H. Long L. (2004). Statistical remedies for common method biases. Adv. Psychol. Sci. 6, 942–950.

