# Supplementary material for: The relationship between self-compassion, coping style, sleep quality, and depression among college students
Source: Front Psychol. 2024 Jun 7;15:1378181. doi: 10.3389/fpsyg.2024.1378181 (PMC11190380; doi:10.3389/fpsyg.2024.1378181)
Supplement: Supplementary file 1 [file Data_Sheet_1.ZIP › Data/A blank copy of the questionnaire(sú⌐.docx]

**大学生生活方式调查**

亲爱的同学：

       您好！我们是山东大学体育学院的研究团队，诚邀您参加【大学生生活方式调查】的研究，本次问卷均采用匿名调查，结果仅供科学研究使用，您的回答只用于统计及相关分析。您只需认真阅读每个题目，根据自己的实际情况，以第一反应真实作答。

       衷心感谢您的支持和帮助！

**一、基本信息**

1. 你的性别： [单选题] *

| ○男 |
| --- |
| ○女 |

2. 你的年龄： [填空题] *

（例如：25岁）

_________________________________

3. 您的专业方向属于： [单选题] *

| ○文科类 |
| --- |
| ○理科类 |
| ○工科类 |
| ○医学类 |

4. 即将进入或所处的学习阶段： [单选题] *

| ○大一 |
| --- |
| ○大二 |
| ○大三 |
| ○大四或大五 |
| ○研究生 |

5. 你目前所在的省份地区与城市： [填空题] *

（例如：山东济南）

_________________________________

6. 是否已返校： [单选题] *

| ○是 |
| --- |
| ○否 |

7. 是否为独生子女 [单选题] *

| ○是 |
| --- |
| ○否 |

8. 家庭所在地 [单选题] *

| ○城镇 |
| --- |
| ○农村 |

9. 相对于其他同学，你家的经济情况如何？ [单选题] *

| ○很差 |
| --- |
| ○较差 |
| ○一般 |
| ○较好 |
| ○很好 |

10. 你的父亲学历? [单选题] *

| ○小学或以下 |
| --- |
| ○初中 |
| ○高中 |
| ○专科 |
| ○本科 |
| ○研究生 |

11. 你的母亲学历? [单选题] *

| ○小学或以下 |
| --- |
| ○初中 |
| ○高中 |
| ○专科 |
| ○本科 |
| ○研究生 |

12. 你父母的婚姻状况如何？ [单选题] *

| ○很和睦 |
| --- |
| ○和睦 |
| ○有时闹矛盾 |
| ○常闹矛盾 |
| ○已分居 |
| ○已离婚 |
| ○父母一方去世 |

13. 你是否有过轻生的念头？ [单选题] *

| ○是 |
| --- |
| ○否 |

14. 过去3个月内你是否有过轻生的念头？ [单选题] *

| ○是 |
| --- |
| ○否 |

**二、本调查表旨在调查人们在日常生活中的体力活动情况。本调查表中的问题是针对您在过去7天中，进行各种体力活动的时间，包括工作交通行程、家务劳动、以及闲暇时间体育锻炼、娱乐活动中的各项体力活动。在回答下面的问题时，请只考虑那些每次至少10分钟的体力活动。**

15. 最近7天内,您有几天做了剧烈的体育活动,像是提重物、挖掘、有氧运动或是快速骑车? [单选题] *

| ○1天 (请跳至第16题) |
| --- |
| ○2天 (请跳至第16题) |
| ○3天 (请跳至第16题) |
| ○4天 (请跳至第16题) |
| ○5天 (请跳至第16题) |
| ○6天 (请跳至第16题) |
| ○7天 (请跳至第16题) |
| ○无相关体育活动→跳到17题 (请跳至第17题) |

16. 在这其中一天您通常会花多少时间在剧烈的体育活动上？

______ 小时 ___ 分钟

[填空题] *

17. 最近7天内，您有几天做了适度的体育活动，像是提轻的物品、以平常的速度骑车或打双人网球? [单选题] *

| ○1天 (请跳至第18题) |
| --- |
| ○2天 (请跳至第18题) |
| ○3天 (请跳至第18题) |
| ○4天 (请跳至第18题) |
| ○5天 (请跳至第18题) |
| ○6天 (请跳至第18题) |
| ○7天 (请跳至第18题) |
| ○无适度体育活动→跳到问题19 (请跳至第19题) |

18. 在这其中一天您通常会花多少时间在适度的体育活动上？

每天______ 小时 ___分钟 [填空题] *

19. 最近7天内，您有几天是步行，且一次步行至少 10分钟? [单选题]

（例如：3天）

| ○1天 _________________ (请跳至第20题) |
| --- |
| ○2天 (请跳至第20题) |
| ○3天 (请跳至第20题) |
| ○4天 (请跳至第20题) |
| ○5天 (请跳至第20题) |
| ○6天 (请跳至第20题) |
| ○7天 (请跳至第20题) |
| ○没有步行→跳到问题21 (请跳至第21题) |

20. 在这其中一天您通常会花多少时间在步行上？

每天 _____ 小时 ___分钟 [填空题] *

21. 最近七天内，工作日您有多久时间是坐着的？

工作日每天_______ 小时 ___分钟 [填空题] *

22. 最近七天内，周末您有多久时间是坐着的？

周末每天_______ 小时 ___分钟 [填空题] *

**三、下面的一些问题是关于你最近1个月的睡眠状况的，请填写或选择最符合你实际情况的答案。**

23. 近一个月，晚上上床睡觉通常几点钟 [单选题] *

| ○22 |
| --- |
| ○23 |
| ○24 |
| ○1 |
| ○2 |
| ○3 |
| ○其他 _________________ |

24. 近一个月，每晚从上床到入睡通常需要多少分钟 [单选题] *

| ○0-15 |
| --- |
| ○16-30 |
| ○31-60 |
| ○61-∞ |

25. 近一个月，每天早上通常几点起床 [单选题] *

| ○6 |
| --- |
| ○7 |
| ○8 |
| ○9 |
| ○10 |
| ○11 |
| ○12 |
| ○其他 _________________ |

26. 近一个月，每晚实际睡眠约几个小时（注意：不等于卧床时间） [单选题] *

| ○4 |
| --- |
| ○5 |
| ○6 |
| ○7 |
| ○8 |
| ○9 |
| ○其他 _________________ |

27. 入睡困难(30分钟内不能入睡) [单选题] *

| ○无 |
| --- |
| ○＜1次/周 |
| ○1-2次/周 |
| ○≥3次/周 |

28. 夜间易醒或早醒 [单选题] *

| ○无 |
| --- |
| ○＜1次/周 |
| ○1-2次/周 |
| ○≥3次/周 |

29. 夜间起床去厕所 [单选题] *

| ○无 |
| --- |
| ○＜1次/周 |
| ○1-2次/周 |
| ○≥3次/周 |

30. 出现呼吸不畅 [单选题] *

| ○无 |
| --- |
| ○＜1次/周 |
| ○1-2次/周 |
| ○≥3次/周 |

31. 咳嗽或鼾声高 [单选题] *

| ○无 |
| --- |
| ○＜1次/周 |
| ○1-2次/周 |
| ○≥3次/周 |

32. 感觉太冷 [单选题] *

| ○无 |
| --- |
| ○＜1次/周 |
| ○1-2次/周 |
| ○≥3次/周 |

33. 感觉太热 [单选题] *

| ○无 |
| --- |
| ○＜1次/周 |
| ○1-2次/周 |
| ○≥3次/周 |

34. 做噩梦 [单选题] *

| ○无 |
| --- |
| ○＜1次/周 |
| ○1-2次/周 |
| ○≥3次/周 |

35. 感到疼痛 [单选题] *

| ○无 |
| --- |
| ○＜1次/周 |
| ○1-2次/周 |
| ○≥3次/周 |

36. 其他影响睡眠的事情 [单选题] *

| ○无 |
| --- |
| ○＜1次/周 |
| ○1-2次/周 |
| ○≥3次/周 |

37. 近一个月，总的来说，您认为自己的睡眠质量 [单选题] *

| ○很好 |
| --- |
| ○较好 |
| ○较差 |
| ○很差 |

38. 近一个月，您用药物催眠的情况 [单选题] *

| ○无 |
| --- |
| ○＜1次/周 |
| ○1-2次/周 |
| ○≥3次/周 |

39. 近一个月，您常感到困倦，难以保持清醒状态吗 [单选题] *

| ○无 |
| --- |
| ○＜1次/周 |
| ○1-2次/周 |
| ○≥3次/周 |

40. 近一个月，您做事情的精力不足吗 [单选题] *

| ○没有 |
| --- |
| ○偶尔有 |
| ○有时有 |
| ○经常有 |

**四、从现在开始向过去推算，近一个月你的心情如何？**

41. 按要求回答以下问题[矩阵量表题] *

|  | 不是 | 有点是 | 中等是 | 基本是 | 是 |
| --- | --- | --- | --- | --- | --- |
| 容易紧张和着急 | ○ | ○ | ○ | ○ | ○ |
| 吃得比过去少 | ○ | ○ | ○ | ○ | ○ |
| 希望痛哭一场 | ○ | ○ | ○ | ○ | ○ |
| 头脑没有平常那样清楚 | ○ | ○ | ○ | ○ | ○ |
| 感到孤独 | ○ | ○ | ○ | ○ | ○ |
| 觉得做人越来越没有意思 | ○ | ○ | ○ | ○ | ○ |
| 对将来不抱有希望 | ○ | ○ | ○ | ○ | ○ |
| 比过去注意力（记忆力）有下降 | ○ | ○ | ○ | ○ | ○ |
| 觉得闷闷不乐，情绪低沉 | ○ | ○ | ○ | ○ | ○ |
| 感到担忧 | ○ | ○ | ○ | ○ | ○ |
| 相信即使个人努力，也不能获得成功 | ○ | ○ | ○ | ○ | ○ |
| 与异性密切接触时，兴趣减少 | ○ | ○ | ○ | ○ | ○ |
| 因为一阵阵头晕而苦恼 | ○ | ○ | ○ | ○ | ○ |
| 觉得自己可能要发疯 | ○ | ○ | ○ | ○ | ○ |
| 容易生气和发火 | ○ | ○ | ○ | ○ | ○ |
| 抽烟或饮酒增加 | ○ | ○ | ○ | ○ | ○ |
| 懒得活动 | ○ | ○ | ○ | ○ | ○ |
| 坐立不安，自己也不知道该做什么 | ○ | ○ | ○ | ○ | ○ |
| 因为头痛、颈痛或背痛而苦恼 | ○ | ○ | ○ | ○ | ○ |
| 容易衰弱和疲乏 | ○ | ○ | ○ | ○ | ○ |
| 如果有可能，真想砸碎一些东西 | ○ | ○ | ○ | ○ | ○ |
| 睡眠比以往差 | ○ | ○ | ○ | ○ | ○ |
| 心烦意乱或觉得惊慌 | ○ | ○ | ○ | ○ | ○ |
| 觉得自己是个无用的人，没人需要我 | ○ | ○ | ○ | ○ | ○ |
| 感到害怕 | ○ | ○ | ○ | ○ | ○ |
| 过去感兴趣的事现在没有兴趣 | ○ | ○ | ○ | ○ | ○ |
| 责怪自己 | ○ | ○ | ○ | ○ | ○ |
| 神经过敏，心中不踏实 | ○ | ○ | ○ | ○ | ○ |

**五、以下是您过去一周内可能有的个体情绪体验或躯体症状。右侧显示的数字表示不同的程度，请您根据自己实际感受和行为情况，选择相应的符合程度。**

42. 按要求回答以下问题[矩阵量表题] *

|  | 不符合 | 有时符合 | 常常符合 | 总是符合 |
| --- | --- | --- | --- | --- |
| 我觉得很难让自己安静下来 | ○ | ○ | ○ | ○ |
| 我感到口干舌燥 | ○ | ○ | ○ | ○ |
| 我好像一点都没有感觉到任何愉快、舒畅 | ○ | ○ | ○ | ○ |
| 我感到呼吸困难（例如：气喘或透不过来气） | ○ | ○ | ○ | ○ |
| 我感到很难主动去开始工作 | ○ | ○ | ○ | ○ |
| 我对事情往往做出过敏反应 | ○ | ○ | ○ | ○ |
| 我感到颤抖（例如，手抖） | ○ | ○ | ○ | ○ |
| 我觉得自己消耗了很多精力 | ○ | ○ | ○ | ○ |
| 我担心一些可能让自己恐慌或者出丑的场合 | ○ | ○ | ○ | ○ |
| 我觉得自己对不久的将来没有什么可期盼的 | ○ | ○ | ○ | ○ |
| 我感到忐忑不安 | ○ | ○ | ○ | ○ |
| 我感到很难放松自己 | ○ | ○ | ○ | ○ |
| 我感到忧郁沮丧 | ○ | ○ | ○ | ○ |
| 我无法容忍任何阻碍我继续工作的事情 | ○ | ○ | ○ | ○ |
| 我感到快要崩溃了 | ○ | ○ | ○ | ○ |
| 我对任何事情都不能产生热情 | ○ | ○ | ○ | ○ |
| 我觉得自己不怎么配做人 | ○ | ○ | ○ | ○ |
| 我发觉自己很容易被触怒 | ○ | ○ | ○ | ○ |
| 即使在没有很明显的体力活动时，我也感到心率不正常 | ○ | ○ | ○ | ○ |
| 我无缘无故的感到害怕 | ○ | ○ | ○ | ○ |
| 我感到生命毫无意义 | ○ | ○ | ○ | ○ |

六、请您根据自己实际感受和行为情况，选择相应的符合程度。

43. 请按要求以下回答[矩阵量表题] *

|  | 非常不符合 | 较不符合 | 有点不符合 | 一般 | 较符合 | 符合 | 非常符合 |
| --- | --- | --- | --- | --- | --- | --- | --- |
| 当我处在积极情绪里的时候，周围的人能很明显的看出来 | ○ | ○ | ○ | ○ | ○ | ○ | ○ |
| 有时看悲伤的电影会令我哭出来 | ○ | ○ | ○ | ○ | ○ | ○ | ○ |
| 别人不容易看出我的情绪状况 | ○ | ○ | ○ | ○ | ○ | ○ | ○ |
| 如果别人讲了个我认为有意思的笑话，我会大声笑出来 | ○ | ○ | ○ | ○ | ○ | ○ | ○ |
| 对我来讲，很难去掩饰恐惧或者害怕的情绪 | ○ | ○ | ○ | ○ | ○ | ○ | ○ |
| 当我高兴快乐的时候就会表现出来 | ○ | ○ | ○ | ○ | ○ | ○ | ○ |
| 我的身体会随着情绪产生很大的反应 | ○ | ○ | ○ | ○ | ○ | ○ | ○ |
| 我觉得学会把怒气压抑下来比把怒气表现出来好 | ○ | ○ | ○ | ○ | ○ | ○ | ○ |
| 不论多么紧张和烦乱，我都会尽力让自己保持一种冷静的形象 | ○ | ○ | ○ | ○ | ○ | ○ | ○ |
| 我是一个情绪上富于表达的人 | ○ | ○ | ○ | ○ | ○ | ○ | ○ |
| 我有着强烈的情绪 | ○ | ○ | ○ | ○ | ○ | ○ | ○ |
| 有时候我藏不住自己的情绪，尽管我很想掩藏起来 | ○ | ○ | ○ | ○ | ○ | ○ | ○ |
| 当我处在负面情绪里的时候，周围的人能很明显的看出来 | ○ | ○ | ○ | ○ | ○ | ○ | ○ |
| 有几次我努力控制自己不要哭，但还是没办法停下来 | ○ | ○ | ○ | ○ | ○ | ○ | ○ |
| 我对自己情绪的体验非常强烈 | ○ | ○ | ○ | ○ | ○ | ○ | ○ |
| 我的情绪感受都会在脸上表现出来 | ○ | ○ | ○ | ○ | ○ | ○ | ○ |

**七、请仔细阅读每个题目，并根据自己的实际情况选择相应选项。答案没有对错之分，请你根据第一反应如实作答，谢谢你的合作。**

44. 按要求回答以下问题[矩阵量表题] *

|  | 几乎不这样 | 很少这样 | 有时这样 | 经常这样 | 几乎总是这样 |
| --- | --- | --- | --- | --- | --- |
| 对自己的缺点和不足，我持不满和批判的态度 | ○ | ○ | ○ | ○ | ○ |
| 情绪低落时，我容易纠结于不顺心的事情 | ○ | ○ | ○ | ○ | ○ |
| 遇到困难时，我把困难看成是生活的一部分，是每个人都会经历的 | ○ | ○ | ○ | ○ | ○ |
| 当我想到自己的缺点时，我容易觉得愈发孤立、与世隔绝 | ○ | ○ | ○ | ○ | ○ |
| 当我心情不好时，我会更关爱自己 | ○ | ○ | ○ | ○ | ○ |
| 当我在一些对自己重要的事情上失败后，我会不断地想自己的不足 | ○ | ○ | ○ | ○ | ○ |
| 当我倒霉的时候，我会提醒自己：其实这世上有很多人和我一样不走运 | ○ | ○ | ○ | ○ | ○ |
| 处境艰难时，我通常会对自己很苛刻 | ○ | ○ | ○ | ○ | ○ |
| 遇到烦心事，我会尽量想办法让自己情绪保持稳定 | ○ | ○ | ○ | ○ | ○ |
| 当我感到自己在某些方面不足时，我尽量提醒自己大部分人和我一样都不完美 | ○ | ○ | ○ | ○ | ○ |
| 对于我性格中那些自己不喜欢的方面，我不能容忍 | ○ | ○ | ○ | ○ | ○ |
| 当我经历苦难时，我会关心、善待自己 | ○ | ○ | ○ | ○ | ○ |
| 当情绪低落时，我会觉得大多数人可能比我快乐 | ○ | ○ | ○ | ○ | ○ |
| 当一些令人痛苦的事情发生时，我尽量用平和的心态来面对 | ○ | ○ | ○ | ○ | ○ |
| 我尽量把自己的失败看成人生经历的一部分 | ○ | ○ | ○ | ○ | ○ |
| 当我意识到自身的缺点时，我会对自己失去信心 | ○ | ○ | ○ | ○ | ○ |
| 在一些对自己重要的事情上失败时，我会尽量全面、客观地认识这些事情 | ○ | ○ | ○ | ○ | ○ |
| 当我很努力去争取某样东西时我觉得其他人得到同样的东西一定会比我轻松些 | ○ | ○ | ○ | ○ | ○ |
| 经历困苦时，我会善待自己 | ○ | ○ | ○ | ○ | ○ |
| 当某些事使我心烦时，我容易受情绪控制而失去理智 | ○ | ○ | ○ | ○ | ○ |
| 经历困苦时，我对自己有点冷酷无情(不善待自己) | ○ | ○ | ○ | ○ | ○ |
| 当情绪低落时，我试图用好奇与开放的心态去面对它 | ○ | ○ | ○ | ○ | ○ |
| 对自己的缺点和不足，我持宽容态度 | ○ | ○ | ○ | ○ | ○ |
| 当一些痛苦的事情发生时，我会夸大它的影响 | ○ | ○ | ○ | ○ | ○ |
| 在一些对自己重要的事情上失败时，我容易觉得是自己一个人在承受失败，感到孤独 | ○ | ○ | ○ | ○ | ○ |
| 我尽量去理解和包容自己性格中自己不喜欢的方面 | ○ | ○ | ○ | ○ | ○ |

**八、当您遇到压力或烦恼时,请选择您采取的应对方式，并选择符合自己情况的选项。**

45. 按要求回答以下问题[矩阵量表题] *

|  | 不采用 | 偶尔采用 | 有时采用 | 经常采用 |
| --- | --- | --- | --- | --- |
| 通过工作学习或一些其他活动解脱 | ○ | ○ | ○ | ○ |
| 尽量想到事物好的一面 | ○ | ○ | ○ | ○ |
| 与人交谈，倾诉内心烦恼 | ○ | ○ | ○ | ○ |
| 改变自己的想法，重新发现生活中什么重要 | ○ | ○ | ○ | ○ |
| 不把问题看得太严重 | ○ | ○ | ○ | ○ |
| 坚持自己的立场，为自己想得到的斗争 | ○ | ○ | ○ | ○ |
| 找出几种不同的解决问题的方法 | ○ | ○ | ○ | ○ |
| 向亲戚朋友或同学寻求建议 | ○ | ○ | ○ | ○ |
| 改变原来的一些做法或自己的一些问题 | ○ | ○ | ○ | ○ |
| 借鉴他人处理类似困难情景的办法 | ○ | ○ | ○ | ○ |
| 寻求业余爱好，积极参加文体活动 | ○ | ○ | ○ | ○ |
| 尽量克制自己的失望、悔恨、悲伤和愤怒 | ○ | ○ | ○ | ○ |
| 通过吸烟、喝酒、服药和吃东西来解除烦恼 | ○ | ○ | ○ | ○ |
| 认为时间会改变现状，唯一要做的便是等待 | ○ | ○ | ○ | ○ |
| 试图休息或休假，暂时把问题（烦恼）抛开 | ○ | ○ | ○ | ○ |
| 试图忘记整个事情 | ○ | ○ | ○ | ○ |
| 依靠别人解决问题 | ○ | ○ | ○ | ○ |
| 接受现实，因为没有其它办法 | ○ | ○ | ○ | ○ |
| 幻想可能会发生某种奇迹改变现状 | ○ | ○ | ○ | ○ |
| 自己安慰自己 | ○ | ○ | ○ | ○ |
